# P60 Investigating *Clostridioides difficile* infection caused by an emerging virulent strain in individual and pooled human microbiomes using MiGut, an *in vitro* gut model

**DOI:** 10.1093/jacamr/dlag102.066

**Published:** 2026-06-26

**Authors:** Nia Paddison Rees, William Davis Birch, Emma Clark, Karen Bentley, Chichi Chogugudza, Nik Kapur, Ines Moura

**Affiliations:** Healthcare Associated Infections Research Group, University of Leeds, Leeds, UK; NIHR Leeds Biomedical Research Centre, Leeds, UK; Healthcare Associated Infections Research Group, University of Leeds, Leeds, UK; School of Mechanical Engineering, University of Leeds, Leeds, UK; Healthcare Associated Infections Research Group, University of Leeds, Leeds, UK; NIHR Leeds Biomedical Research Centre, Leeds, UK; Healthcare Associated Infections Research Group, University of Leeds, Leeds, UK; Healthcare Associated Infections Research Group, University of Leeds, Leeds, UK; School of Mechanical Engineering, University of Leeds, Leeds, UK; Healthcare Associated Infections Research Group, University of Leeds, Leeds, UK

## Abstract

**Background:**

Antibiotic-induced perturbations in the gut microbiome result in weakened colonization resistance conferred by endogenous bacteria. During dysbiosis, pathogens such as *Clostridioides difficile* can proliferate, causing toxin-mediated damage to colonocytes. *C. difficile* infection (CDI) is the principal cause of healthcare-associated diarrhoea and is a considerable threat due to its status as an MDR organism and the emergence of new epidemic strains. *In vitro* human gut models have been extensively utilized to study CDI induction, dynamics and treatments. However, their large size and low throughput hinders the study of individual microbiomes, making it difficult to predict personalized intervention responses.

**Objective:**

We aimed to test the impact of different antibiotics on individual and pooled microbiomes, and each drug potential to induce simulated CDI by the newly identified epidemic *C. difficile* PCR ribotype 955 (RT955) strain.

**Methods:**

Twelve MiGut *in vitro* models were inoculated in triplicate with three individual donor microbiomes (>55 years age) and a pooled microbiome of all three. All models were dosed with RT955 spores and instilled with either moxifloxacin, ceftriaxone or clindamycin. Models were monitored thrice weekly for bacterial dynamics, spore germination and viable cell proliferation coinciding with toxin production.

**Results:**

CDI was successfully induced in all except two of the models. Whilst the pooled microbiomes experienced CDI post-antibiotics, CDI onset was delayed concurrent with lower *C. difficile* toxin levels compared with the individual donors. Ceftriaxone and moxifloxacin dosing failed to induce simulated CDI in individual donors 1 and 3, respectively.

**Conclusions:**

Induction of simulated CDI was donor-specific, highlighting the importance of single-donor *in vitro* models in informing personalized antimicrobial chemotherapy by demonstrating the impact of different antibiotics on the microbiome. Further RT955 phenotypic characterization is needed alongside efficacy assessment of the current CDI treatment options.

